# Social Risk Prevalence in Adolescent and Young Adult Patients With and Without a History of Cancer

**DOI:** 10.1001/jamanetworkopen.2026.0244

**Published:** 2026-03-02

**Authors:** Carol Y. Ochoa-Dominguez, David M. Mosen, Kimberly A. Miller, Randall Y. Chan, John F. Dickerson, Erin Keast, Matthew P. Banegas

**Affiliations:** 1 Department of Radiation Medicine and Applied Sciences, University of California, San Diego, La Jolla; 2 Center for Health Equity Education and Research, University of California, San Diego, La Jolla; 3 Kaiser Permanente Center for Health Research, Kaiser Permanente Northwest, Portland, Oregon; 4 Department of Population and Public Health Sciences, Keck School of Medicine, University of Southern California, Los Angeles; 5 Department of Dermatology, Keck School of Medicine, University of Southern California, Los Angeles; 6 Los Angeles General Medical Center, Los Angeles, California; 7 Department of Pediatrics, Keck School of Medicine, University of Southern California, Los Angeles

## Abstract

**Importance:**

Health care practices rarely assess or assist patients with social risks, thereby missing a crucial aspect of medical care.

**Objective:**

To assess the prevalence of social risks among adolescents and young adults (AYAs) with and without a history of cancer within an integrated US health system.

**Design, Setting, and Participants:**

This cross-sectional study analyzed data from AYA (aged 15-40 years) members of Kaiser Permanente Northwest (Oregon and Washington) who completed a social risk screener between January 1, 2022, and December 31, 2024.

**Exposure:**

Cancer history identified via electronic health records.

**Main Outcomes and Measures:**

Four patient-reported social risk domains (financial hardship, food insecurity, housing instability, and transportation difficulties) were assessed as binary (yes or no) indicators. A composite binary variable was created to reflect the presence or absence of any social risk. Descriptive statistics and multivariable logistic regression models were used to compare prevalence and identify associated factors.

**Results:**

Of 96 127 AYA patients (6.2% aged 15-19 years, 37.4% aged 20-29 years, and 56.4% aged 30 to 40 years; 63.3% female; 13.0% identifying as Hispanic, 63.4% as non-Hispanic White, and 23.6% as non-Hispanic other race and ethnicity), 1239 (1.3%) had a history of cancer, and these patients compared with those without cancer were older (aged 30-40 years, 82.6% vs 56.0%), were more likely to be female (81.4% vs 63.1%) and non-Hispanic White (74.3% vs 63.3%), and had more comorbidities (8.8% vs 3.9%). No significant differences were observed in household income, education level, or neighborhood deprivation between groups. Thirty percent of patients with cancer and 33% without cancer reported experiencing at least 1 social risk. Financial hardship was the most common issue, followed by food insecurity, housing instability, and transportation difficulties. Higher odds of social risks were observed among patients aged 20 to 29 years (odds ratio [OR], 1.58 [95% CI, 1.54-1.63]) and those with 3 or more comorbidities (OR, 1.79 [95% CI, 1.67-1.92]), prior medical financial assistance (OR, 2.10 [95% CI 1.99-2.22]), or identifying as Hispanic (OR, 1.14 [95% CI, 1.09-1.19]). Lower odds were found among female patients (OR, 0.91 [95% CI, 0.88-0.94]), commercially insured patients (OR, 0.32 [95% CI, 0.31-0.34]), longer Kaiser Permanente Northwest membership (OR, 0.99 [95% CI, 0.99-0.99]), and residents of less-deprived neighborhoods (neighborhood deprivation index [NDI] quartile 1: OR, 0.65 [95% CI, 0.62-0.67]; NDI quartile 2: OR, 0.74 [95% CI, 0.71-0.77]; NDI quartile 3: OR, 0.81; 95% CI, 0.78-0.84).

**Conclusions and Relevance:**

This cross-sectional study found that regardless of cancer history, AYA patients may experience a high burden of social risks. These findings support the need to routinely assess and address social risks in AYA populations to improve equity in health care. Future research should investigate the association of social risks with health care use and long-term outcomes.

## Key Points

**Question:**

What is the prevalence of social risks among adolescent and young adult patients with and without cancer?

**Findings:**

In this cross-sectional study of 96 127 patients, 30% of those with cancer and 33% without cancer reported at least 1 social risk, with financial hardship being most common. Social risk was higher among patients aged 20 to 29 years and those with multiple comorbidities, prior medical financial assistance, or identifying as Hispanic, and a lower risk was observed among female patients, those with commercial insurance and longer health system membership, and residents of less-deprived areas.

**Meaning:**

These findings reveal the magnitude of social risks among adolescent and young adult patients and emphasize the importance of routine screening to reduce disparities and promote equitable care.

## Teaser

This cross-sectional study assesses the prevalence of social risks among adolescents and young adults (AYAs) with and without a history of cancer within an integrated US health system.

## Introduction

Social risks, such as financial hardship, food insecurity, housing instability, and transportation difficulties, are highly prevalent across the US and are associated with poor health outcomes.^1^ An estimated 13.5% of households (18.0 million) experience food insecurity, while 5.7% of adults (13.0 million) report lacking reliable transportation.^2,3^ Additionally, 42.0 million households are cost-burdened, spending more than one-third of their income on housing.^4^ Medical financial hardship affects 56.0% of adults (137.1 million), and the number of individuals delaying medical care due to transportation barriers increased from 4.8 million in 1997 to 5.8 million in 2017.^5,6^

While these challenges are well documented in the general population, few studies have explored the prevalence and associations of social risks among adolescents and young adults (AYAs) (aged 15-39 years), a group with distinct developmental, financial, and health care needs that increase susceptibility to social risks. Population-based studies have indicated that food insecurity, housing instability, and transportation barriers are not only common in this age group but also associated with self-reported chronic disease and obesity.^4,7,8,9^ For AYA cancer survivors, these challenges are often magnified by the physical, emotional, and financial burdens of cancer diagnosis and treatment.^10,11,12,13,14^ While prior research in this population has largely focused on employment and financial hardship, other dimensions of social risk, such as food insecurity, housing instability, and transportation barriers, remain understudied.^15,16,17^ However, financial hardship often intersects with and may exacerbate these social risks, which may lead to delayed or forgoing medical care, difficulties adhering to treatment and follow-up recommendations, and increased mortality risk.^18,19^ The economic toll of cancer is substantial: AYAs face disproportionately high treatment costs, and estimates have shown that their annual productivity loss is more than twice that of peers without a cancer history.^20,21^

In this study, we leveraged unique data from Kaiser Permanente Northwest (KPNW), including social risk data linked to comprehensive electronic health records, to examine and compare the prevalence of social risks among AYA patients with and without a history of cancer. Understanding differences in social risk burden between these groups is important because AYA patients with cancer may have distinct opportunities for identification and intervention through cancer-specific clinical workflows, survivorship programs, and supportive care resources that are not routinely available to patients without a cancer history. This study aimed to characterize and compare the prevalence of social risks among AYA patients with and without a history of cancer.

## Methods

### Data Source, Study Design, and Study Population

This cross-sectional study used data from KPNW, an integrated health care system serving more than 620 000 members in northwest Oregon and southwest Washington, representing approximately 16% of the region’s population. The KPNW Institutional Review Board deemed the study exempt from review and informed consent under category 4 of the Common Rule, meaning that this research was determined to be low risk as it involves the use of secondary data. The study followed the Strengthening the Reporting of Observational Studies in Epidemiology (STROBE) reporting guideline for cross-sectional studies.

Members of KPNW are demographically similar to the surrounding community, with a broad age distribution (approximately 23% aged 18-35 years and 40% aged 36-64 years), a nearly equal sex distribution (52% female), and a racial and ethnic composition comparable to that of the regional population. Approximately 18% of members live below 200% of the federal poverty level, and approximately 80% receive coverage through employer-sponsored plans, contributing to high annual retention rates (approximately 88%). In early 2020, KPNW implemented a social risk screening tool via the EPIC-based (Epic Systems Corp) HealthConnect system. Full details of the screening process have been previously described.^22,23^

We included KPNW members aged 15 to 40 years who (1) received care at KPNW; (2) completed the social determinants of health screener between January 1, 2022, and December 31, 2024 (the first screening was defined as the index date); and (3) had at least 6 months of follow-up data (eFigure 1 in Supplement 1).

### Measures

The primary independent variable was cancer history, defined by electronic health record documentation of a primary cancer diagnosis. The outcome was a binary variable indicating whether social risk was present (yes or no), defined as reporting at least 1 of the following domains: financial hardship, food insecurity, housing instability, or transportation difficulties. We used established measures and methods that have been applied in prior studies (eMethods in Supplement 1).^24^ We used the first reported social risk assessment for these analyses.

We extracted variables from the electronic health record at the patient, clinic, and community levels. Patient demographics included sex (female or male), self-reported race and ethnicity collapsed into 3 categories (Hispanic, non-Hispanic White, and non-Hispanic other [American Indian or Alaska Native, Asian, Black, other, unknown]) to ensure adequate sample sizes and analytic stability while capturing key racial and ethnic disparities relevant to social risk, age (at the time for social risk screening and time at diagnosis), Elixhauser comorbidity score, and health insurance at the time of social risk screening (commercial, Medicaid, Medicare, other, and multiple health insurances). The unweighted Elixhauser comorbidity score without cancer was used, which measures patient comorbidity based on *International Classification of Diseases, Ninth Revision, Clinical Modification* and *International Statistical Classification of Diseases, Tenth Revision* diagnoses.^25^ For patients with cancer, we also included time since diagnosis, cancer site, and tumor stage. Cancer sites were categorized as reproductive system (breast, uterine cervix, corpus and uterus, and male genital system), solid tumors (colon and rectum, skin, endocrine, brain and other nervous system, digestive, eye and orbit, bone and soft tissue, oral cavity and pharynx, respiratory, urinary, and unknown), and hematologic cancers (leukemia and lymphoma). Clinic-level variables included survey response year (2022-2024), duration of KPNW membership, and receipt of prior medical financial assistance from KPNW before social risk screening (yes or no). Community-level variables were derived from the 2020 American Community Survey and included median household income; educational attainment (tract-level high school diploma or less); and the neighborhood deprivation index (NDI), which summarizes socioeconomic context across 13 US census–based indicators covering poverty, occupation, housing, income, employment, and education, with a higher score indicating higher neighborhood deprivation.^26,27^

### Statistical Analysis

All analyses were conducted using SAS, version 9.4 (SAS Institute Inc). We compared characteristics by cancer history using *t* and χ^2^ tests and reported the prevalence of social risks overall and by cancer history. We used logistic regression to examine the association between cancer history and any social risk, adjusting for the following key factors: survey year, age, comorbidity score, receipt of medical financial assistance, health insurance, sex, 2020 American Community Survey NDI, race and ethnicity, and duration of KPNW membership. We did not include median household income and educational attainment as factors because they would be collinear with NDI, which encompasses income and education indicators. To assess cancer-specific associations with social risk, we restricted analyses to patients with cancer and modeled cancer-specific variables (eg, time since diagnosis, cancer site, stage). Several models were estimated, and model fit was statistically evaluated using the criterion described in eTable 1 in Supplement 1.

Sensitivity analyses were performed among patients aged 18 years or older to account for possible caregiver responses among younger AYAs, and results were consistently in the same direction (eTables 2 and 3 in Supplement 1). Missing data were minimal, ranging from 0.02% to 5.00%, and were assumed to be missing completely at random for most variables (excluding Elixhauser comorbidity score) for purposes of analysis. For the Elixhauser comorbidity score, approximately 13% of the population had missing data, mostly among AYA patients without a history of cancer. For this variable, we decided to create a category for the missing data, as we believed that there might be mechanisms for the missingness that we could not observe (eg, access issues, benefit structures). However, it is still important to capture those patients. Additional sensitivity analysis were conducted and can be found in the eMethods and eTables 4 and 5 in Supplement 1.

All tests were 2-sided, with α = .05. We used the Benjamini-Hochberg procedure to control for false discovery, with a maximum false discovery rate of 20%.^28^ We report odds ratios (ORs) and 95% CIs.

## Results

### Comparison of AYA Patients With and Without a History of Cancer

Table 1 presents study sample characteristics. The final sample included 96 127 patients (6.2% aged 15-19 years, 37.4% aged 20-29 years, and 56.4% aged 30 to 40 years; 63.3% female and 36.7% male; 13.0% identifying as Hispanic, 63.4% as non-Hispanic White, and 23.6% as non-Hispanic other race and ethnicity), of whom 1239 (1.3%) had a history of cancer after excluding nonmelanoma skin cancers. Most surveys were completed in 2023 (41.0%). Patients with cancer compared with those without cancer were older (aged 30-40 years, 82.6% vs 56.0%) and more likely to be female (81.4% vs 63.1%) and non-Hispanic White (74.3% vs 63.3%). No significant differences were observed in median household income, the proportion of tracts with a high school diploma or less, or the NDI. Patients with cancer compared with those without cancer were more likely to have received prior medical financial assistance (14.7% vs 6.2%), had more comorbidities (8.8% vs 3.9%), and had a longer KPNW membership duration (mean [SD], 89.3 [85.9] vs 68.4 [72.0] months).

**Table 1.** Characteristics of AYA Patients With and Without a History of Cancer

Variable	Patients, No. (%)			*P *value^a^
	Total sample (N = 96 127)	Without cancer (n = 94 888)	With cancer (n = 1239)	
Survey response year				
2022	30 680 (31.9)	30 173 (31.8)	507 (40.9)	<.001
2023	39 391 (41.0)	38 902 (41.0)	489 (39.5)	
2024	26 056 (27.1)	25 813 (27.2)	243 (19.6)	
Age at social risk screening, y				
15-19	5957 (6.2)	5930 (6.3)	27 (2.2)	<.001
20-29	35 938 (37.4)	35 749 (37.7)	189 (15.2)	
30-40	54 232 (56.4)	53 209 (56.0)	1023 (82.6)	
Sex				
Female	60 783 (63.3)	59 775 (63.1)	1008 (81.4)	<.001
Male	35 201 (36.7)	34 970 (36.9)	231 (18.6)	
Race and ethnicity				
Hispanic	12 499 (13.0)	12 355 (13.0)	144 (11.6)	<.001
Non-Hispanic White	60 948 (63.4)	60 028 (63.3)	920 (74.3)	
Non-Hispanic other^b^	22 680 (23.6)	22 505 (23.7)	175 (14.1)	
Household income, $				
Mean (SD)	78 497.75 (25 794.53)	78 497.40 (25 802.10)	78 524.80 (25 217.30)	.97
Range	8955 to 218 114	8955 to 218 114	22 574 to 189 545	
High school education or less^c^				
Mean (SD)	0.29 (0.13)	0.29 (0.13)	0.29 (0.13)	.41
Range	0 to 0.79	0 to 0.79	0 to 0.67	
2020 ACS NDI^c^				
Mean (SD)	−0.15 (0.72)	−0.15 (0.72)	−0.14 (0.72)	.57
Range	−1.78 to 2.96	−1.78 to 2.96	−1.71 to 2.86	
Elixhauser comorbidity score				
0	54 626 (56.8)	53 948 (56.9)	678 (54.7)	<.001
1	18 709 (19.4)	18 437 (19.4)	272 (22.0)	
2	6581 (6.9)	6460 (6.8)	121 (9.7)	
≥3	3826 (4.0)	3717 (3.9)	109 (8.8)	
Unknown	12 385 (12.9)	12 326 (13.0)	59 (4.8)	
Health insurance type				
Commercial	81 763 (85.2)	80 733 (85.2)	1030 (83.2)	.046
Other^d^	14 205 (14.8)	13 997 (14.8)	208 (16.8)	
Duration of KPNW membership before screening, mo				
Mean (SD)	68.7 (72.2)	68.4 (72.0)	89.3 (85.9)	<.001
Range	6 to 487	6 to 491	6 to 488	
MFA prescreening				
Yes	6051 (6.3)	5869 (6.2)	182 (14.7)	<.001
No	90 076 (93.7)	89 019 (93.8)	1057 (85.3)	
Age at cancer diagnosis, y				
15-19	NA	NA	62 (5.4)	NA
20-29	NA	NA	445 (38.9)	
30-39	NA	NA	636 (55.6)	
Time since diagnosis, y	NA	NA		
<1	NA	NA	250 (21.3)	NA
1-5	NA	NA	431 (36.7)	
≥5	NA	NA	492 (41.9)	
Cancer site^e^	NA	NA		
Reproductive	NA	NA	589 (47.5)	NA
Solid	NA	NA	563 (45.4)	
Hematologic	NA	NA	87 (7.0)	
Tumor stage	NA	NA		
0	NA	NA	255 (20.6)	NA
1	NA	NA	291 (23.5)	
2	NA	NA	73 (5.9)	
3	NA	NA	81 (6.5)	
4	NA	NA	38 (3.1)	
Unspecified or unstaged^f^	NA	NA	501 (40.4)	

Abbreviations: ACS, American Community Survey; AYA, adolescent or young adult; MFA, medical financial assistance; KPNW, Kaiser Permanente Northwest; NA, not applicable; NDI, neighborhood deprivation index.

^a^ *P* values are 2-sided.

^b^ Included American Indian or Alaska Native, Asian, Black, multiracial, other race, or unknown.

^c^ Educational attainment indicates the US census tract–level proportion of adults with a high school diploma or less, with higher mean values indicating lower educational attainment. The NDI is a census-based composite measure of socioeconomic disadvantage, with higher mean scores indicating greater neighborhood deprivation.

^d^ Included Medicaid, Medicare, other, and multiple health insurance.

^e^ Reproductive included breast, uterine cervix, corpus and uterus, and male genital system; solid tumors included those of the colon and rectum, skin, endocrine system, brain and other nervous system, digestive system, eye and orbit, bones and soft tissues, oral cavity and pharynx, respiratory system, urinary system, digestive system, and unknown; and hematologic cancers included leukemia and lymphoma.

^f^ Stage not assigned at diagnosis or stage information at time of diagnosis not available.

Most patients with cancer were diagnosed between age 30 and 40 years (55.6%), with 38.9% diagnosed between age 20 and 29 years and 5.4% between age 15 and 19 years (Table 1). Regarding the time since diagnosis, 21.3% of patients had been diagnosed within the past year, 36.7% had been diagnosed between 1 and less than 5 years prior, and 41.9% had been diagnosed 5 years or more prior. Reproductive cancers were most common (47.5%), followed by solid tumors (45.4%) and hematologic cancers (7.0%). Tumor stage distribution varied, with 40.4% of patients having an unspecified or unknown stage.

### Prevalence of Social Risks

Patients with cancer reported a lower prevalence of all 4 social risk domains (Figure; eFigure 2 in Supplement 1). Thirty percent of patients with cancer and 33% without cancer reported at least 1 social risk. Financial hardship was most common among both patients with and without cancer (23% vs 25%, respectively), followed by food insecurity (18% vs 20%, respectively), housing instability (11% vs 12%, respectively), and transportation difficulties (6% vs 7%, respectively).

**Figure.**
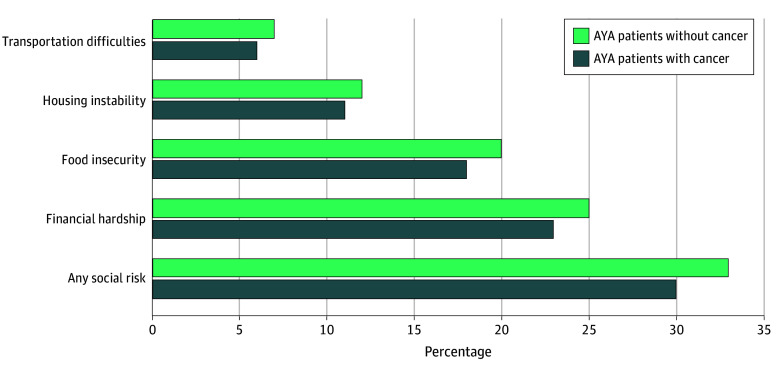
Bar Chart of Prevalence of Social Risks by Adolescent and Young Adult (AYA) Patients With and Without a History of Cancer

### Association Between AYA Cancer History and Any Social Risk

The adjusted model showed that AYA patient cancer history was not significantly associated with social risk (OR, 0.88 [95% CI, 0.78-1.01]) (Table 2; eFigure 3 in Supplement 1). Eight factors were significantly associated with AYAs reporting any social risks. In the adjusted models, patients aged 15 to 19 years had a lower odds of reporting any social risks (OR, 0.86 [95% CI, 0.80-0.91]), while those aged 20 to 29 years had a higher odds of reporting any social risks (OR, 1.58 [95% CI, 1.54-1.63]) compared with patients aged 30 to 40 years. Higher odds were also observed among individuals with 3 or more comorbidities (OR, 1.79 [95% CI, 1.67-1.92]), those who had received prior medical financial assistance (OR, 2.10 [95% CI, 1.99-2.22]), and those of Hispanic ethnicity (OR, 1.14 [95% CI, 1.09-1.19]). Lower odds were found among female patients (OR, 0.91 [95% CI, 0.88-0.94]), commercially insured patients (OR, 0.32 [95% CI, 0.31-0.34]), those living in less-deprived neighborhoods (NDI quartile 1: OR, 0.65 [95% CI, 0.62-0.67]; NDI quartile 2: OR, 0.74 [95% CI, 0.71-0.77]; NDI quartile 3: OR, 0.81 [95% CI, 0.78-0.84]), and those with longer KPNW membership (OR, 0.99 [95% CI, 0.99-0.99]).

**Table 2.** Association Between AYA Cancer History and Any Social Risk

Variable	Any social risk			
	Unadjusted		Adjusted	
	OR (95% CI)	*P* value^a^	OR (95% CI)	*P* value^a^
Cancer (reference, no cancer)	0.87 (0.77-0.99)	.03	0.88 (0.78-1.01)	.06
Survey year (reference, 2024)				
2022	0.95 (0.92-0.98)	.003	0.96 (0.93-1.00)	.06
2023	0.96 (0.93-0.99)	.01	1.00 (0.97-1.04)	.84
Age group (reference, 30-40 y)				
15-19 y	0.80 (0.76-0.86)	<.001	0.86 (0.80-0.91)	<.001
20-29 y	1.54 (1.50-1.59)	<.001	1.58 (1.54-1.63)	<.001
Elixhauser comorbidity score (reference, 0)				
1	1.50 (1.45-1.56)	<.001	1.42 (1.37-1.47)	<.001
2	1.93 (1.83-2.04)	<.001	1.70 (1.61-1.80)	<.001
≥3	2.24 (2.10-2.40)	<.001	1.79 (1.67-1.92)	<.001
Unknown	1.26 (1.21-1.32)	<.001	1.18 (1.13-1.24)	<.001
Received MFA (reference, no MFA)	2.25 (2.14-2.37)	<.001	2.10 (1.99-2.22)	<.001
Commercial insurance (reference, other)	0.29 (0.28-0.30)	<.001	0.32 (0.31-0.34)	<.001
Female sex (reference, male sex)	1.02 (0.99-1.05)	.15	0.91 (0.88-0.94)	<.001
NDI quartile (reference, 4 [most deprived])				
1 (Least deprived)	0.56 (0.54-0.58)	<.001	0.65 (0.62-0.67)	<.001
2	0.66 (0.64-0.69)	<.001	0.74 (0.71-0.77)	<.001
3	0.75 (0.72-0.78)	<.001	0.81 (0.78-0.84)	<.001
Race and ethnicity (reference, non-Hispanic White)				
Hispanic	1.38 (1.33-1.44)	<.001	1.14 (1.09-1.19)	<.001
Non-Hispanic other	1.05 (1.02-1.09)	.002	0.99 (0.96-1.03)	.77
KPNW membership duration	0.99 (0.99-0.99)	<.001	0.99 (0.99-0.99)	<.001

Abbreviations: AYA, adolescent and young adult; OR, odds ratio; KPNW, Kaiser Permanente Northwest; MFA, medical financial assistance; NDI, neighborhood deprivation index.

^a^ The Benjamini-Hochberg procedure was used to control for false discovery, with a maximum false discovery rate of 20%.

### Cancer Characteristics and Social Risks

In adjusted models, 2 significant associations were observed (Table 3). The first association was between time since diagnosis and the odds of reporting any social risks, with patients with cancer between 1 and less than 5 years prior being more likely to report any social risk (OR, 1.47 [95% CI, 1.01-2.13]). Second, cancer site was associated with the odds of reporting any social risks, with patients with hematologic (OR, 1.93 [95% CI, 1.07-3.50]) and reproductive (OR, 1.58 [95% CI, 1.12-2.21]) cancers more likely to report any social risk. Age at diagnosis and tumor stage were not significantly associated with social risks.

**Table 3.** Association of Cancer Risk Factors With Any Social Risk Among AYA Patients With Cancer

Variable	Any social risk			
	Unadjusted		Adjusted	
	OR (95% CI)	*P* value^a^	OR (95% CI)	*P* value^a^
Age at diagnosis (reference, 30-39 y)				
15-19 y	0.93 (0.53-1.65)	.81	1.22 (0.66-2.25)	.52
20-29 y	0.92 (0.71-1.20)	.54	1.09 (0.80-1.47)	.59
Time since diagnosis (reference ≥5 y)				
<1 y	1.14 (0.81-1.59)	.46	1.52 (0.99-2.34)	.057
1 to <5 y	1.38 (1.04-1.83)	.02	1.47 (1.01-2.13)	.04
Cancer site (reference, solid tumors)^b^				
Reproductive	1.28 (0.99-1.65)	.10	1.58 (1.12-2.21)	.009
Hematologic	1.50 (0.93-2.41)	.06	1.93 (1.07-3.50)	.03
Tumor stage (reference, unknown or unstaged)^c^				
0	0.68 (0.49-0.96)	.03	0.84 (0.55-1.28)	.41
1	0.80 (0.58-1.09)	.16	1.07 (0.72-1.60)	.73
2	0.86 (0.51-1.47)	.59	1.01 (0.55-1.84)	.99
3	0.89 (0.54-1.48)	.66	1.18 (0.67-2.10)	.57
4	0.71 (0.34-1.51)	.38	0.68 (0.27-1.70)	.24

Abbreviations: AYA, adolescent and young adults; OR, odds ratio.

^a^ The Benjamini-Hochberg procedure was used to control for false discovery, with a maximum false discovery rate of 20%.

^b^ Reproductive included uterine cervix, corpus and uterus, and male genital system; solid tumors included those of the colon and rectum, skin, endocrine system, brain and other nervous system, digestive system, eye and orbit, bones and soft tissues, oral cavity and pharynx, respiratory system, urinary system, digestive system, and unknown; and hematologic cancers included leukemia and lymphoma.

^c^ Unknown or unstaged included no assigned tumor stage at diagnosis, or stage information at time of diagnosis was unavailable.

## Discussion

This cross-sectional study uncovered novel information about the prevalence of social risks among AYA patients with cancer, a group often overlooked in social risk research despite distinct developmental, financial, and health care needs.^29,30^ Nearly one-third of both patients with and without a history of cancer reported at least 1 social risk, most commonly financial hardship, followed by food insecurity, housing instability, and transportation difficulties. While patients with cancer initially seemed less likely to report social risks, this difference was no longer significant after accounting for demographic, clinical, and socioeconomic factors. Social risk was more common among patients with comorbidities, of a younger age (20-29 years), of Hispanic ethnicity, and in receipt of prior medical financial assistance. Conversely, social risk was less common among female AYAs, those with commercial insurance, those with longer KPNW membership, and residents of less-deprived neighborhoods. Among patients with cancer, being diagnosed between 1 and less than 5 years prior was significant for higher social risk. These findings highlight that although patients with cancer may have greater clinical needs, social risks remain a widespread concern across AYA populations, underscoring the need for routine social risk screening and targeted support for this age group.

Unlike prior studies that primarily focused on financial hardship, our data captured multiple social risk domains.^31,32^ We found slightly lower financial hardship among patients with cancer compared with those without cancer (23% vs 25%), differing from Li et al^31^ who reported higher material (23% vs 15%) and behavioral (34% vs 24%) financial hardship among young adult cancer survivors (aged 18-39 years). Additionally, 20% of AYAs without cancer in our study reported food insecurity, which is almost double the national estimate of 11% for young adults aged 24 to 32 years.^7^ Previous research has shown variability in food insecurity by cancer type, eg, 4.4% among breast cancer survivors vs 9.3% among women without cancer and 17.7% among individuals with head and neck cancer compared with approximately 10% with thyroid and colon cancers.^33,34^ We found no prior studies comparing housing instability between AYAs with and without a history of cancer; however, we have reported lower rates (11% among patients with cancer, 12% among patients without cancer) than previous findings among adult survivors (aged ≥18 years) (16.6%).^35^ As well, transportation barriers were similar in our sample (6% among patients with cancer, 7% among patients without cancer) to national estimates for adults aged 18 to 34 years (7.0%).^4^ The higher prevalence of food insecurity compared with transportation difficulties may reflect the resource availability in health care settings. In prior qualitative work, caregivers commonly referenced transportation support, whereas food-related support was less known or accessible.^36^ As many AYAs in our study fall into the middle-income bracket, they or their families might earn too much to qualify for government food assistance programs but still struggle to afford nutritious food. These findings underscore the value of a population-based approach for social risk screening, suggesting a greater magnitude of the issue than previously reported in the literature. Furthermore, quantifying the prevalence of these social risks may inform screening policies, supportive care programs, and resource allocation.

Although we found no association between cancer history and reporting any social risk after adjustment, it is notable that nearly one-third of all AYAs in our sample reported financial hardship, underscoring the substantial affordability challenges faced by this population overall. This finding aligns with broader evidence that AYAs experience disproportionate financial burden due to lower wages, employment instability, education debt, and limited access to employer-sponsored benefits during key life transitions.^37^ At the policy level, affordability protections have largely focused on Medicare beneficiaries, whereas AYAs more commonly rely on Medicaid or private insurance and may be particularly susceptible to changes in Medicaid eligibility, coverage continuity, and cost-sharing. Anticipated Medicaid policy shifts and ongoing coverage instability may further exacerbate financial hardship and unmet social needs among AYAs.

Despite expectations that cancer would heighten social risk, our findings were unexpected, as cancer diagnosis and survivorship among AYAs are often presumed to intensify social hardships, including financial strain, housing instability, and food insecurity.^38,39^ Several potential explanations may account for this finding. One possibility is underreporting by patients with cancer, who may prioritize medical needs over disclosing social challenges or may not recognize the relevance of available resources. For example, a recent qualitative study that explored the perspectives of patients with cancer, clinicians, and patient navigators found that some patients may not report social needs because “they do not realize what the resources can do” to improve their personal lives or their cancer treatment experiences,^40^ suggesting that patients with cancer may not perceive discussing social needs as relevant or urgent in the context of oncology care. Another plausible explanation is that patients with cancer had longer average KPNW membership and may have been able to afford earlier access to support services (eg, patient navigators). Moreover, they may benefit from informal support networks (family, community, and cancer organizations), which are not captured in clinical data. Although we adjusted for internal medical financial assistance, patients with cancer were twice as likely to have received it, which may have reduced their financial burden and freed resources for basic needs. Together, these findings underscore the complex nature of social risk among AYAs and emphasize the importance of considering both formal and informal support systems in future assessments.

We also found that social risk was more prevalent among AYAs with multiple comorbidities, those aged 20 to 29 years, those with a history of receiving medical financial assistance, and those of Hispanic ethnicity and was less prevalent among female patients, patients with commercial insurance, residents of less-deprived neighborhoods, and patients with longer KPNW membership. Among AYAs with a cancer history, social risk was significantly more prevalent among those diagnosed 1 to less than 5 years prior and among those with hematologic and reproductive cancers. The association between comorbidities and social risk is consistent with existing literature, which links multiple health conditions to more complex care needs, increased health care use, and greater financial burden.^41^ Similarly, younger patients, particularly those aged 20 to 29 years, were at higher risk of experiencing social risks, which may be a result of younger patients transitioning into independent adulthood in which they often do not have stable employment and have limited financial savings or insurance coverage.^39,42^ It is also unsurprising that patients who previously received medical financial assistance were more likely to report social risks, potentially reflecting ongoing financial instability. We also found that patients who self-identified as Hispanic reported more social risk, consistent with prior literature in which individuals of an ethnic minority were found to be more likely to experience social risks.^19,43,44^ Qualitative research has suggested that these risks may be shaped by intersecting structural factors, including low income, immigration-related barriers, and limited access to culturally responsive support services, which may persist throughout the cancer care continuum.^36,43^ Collectively, these findings suggest that AYAs who are younger, are managing multiple health conditions, or have previously needed financial support may face compounded challenges in maintaining employment, managing daily responsibilities, and accessing essential services, factors that contribute to increased social risks. Finally, patients with cancer diagnosed 1 to less than 5 years prior had a higher likelihood of social risk, which may reflect reduced access to informal or external support over time.

### Limitations

Our study had several limitations. First, the data were drawn from a single integrated health system, which may limit the generalizability of the findings to other health care settings or populations (eg, those served by public insurance, those without insurance). Second, our sample size, particularly AYA patients with cancer (n = 1239), may have limited our statistical power to detect more nuanced differences. Third, our dependent outcome was self-reported data, which may be subject to recall bias. Fourth, unmeasured confounding variables may have influenced our results. Fifth, our data were cross-sectional; therefore, we could not infer causality from the observed associations. Finally, we lacked the data necessary to compare demographic characteristics between patients who completed the social risk screening and those who did not, which may introduce bias based on who elected to complete the assessment. Despite these limitations, our findings underscore the crucial need to screen for social risks among AYA populations to inform equitable care and support.

## Conclusions

This cross-sectional study found a high prevalence of social risk among AYAs, regardless of their cancer history. The findings underscore the urgent need for routine social risk screening and support within clinical settings. Health systems and oncology practices might benefit from establishing dedicated pathways to assess and address social risks,^45^ which could include integrating accessible social workers or resource navigators to facilitate early, frequent screening and ensure timely identification and mitigation in this vulnerable population. Importantly, our findings emphasized that social risks were not always predictable and underscored the need for longitudinal studies to better understand their development over time. These patterns challenged assumptions about who was at risk and emphasized the importance of implementing repeated, routine assessments rather than relying on one-time screenings or visible indicators alone. Although patients with cancer in our sample reported slightly lower overall social risk, the presence of multiple comorbidities, commonly observed among patients with complex health conditions such as cancer, was associated with higher social risk. This finding suggests that even if patients with cancer as a group seem less burdened by social risk, those with greater clinical complexity may remain highly susceptible and should not be overlooked in social risk assessments. Taken together, these findings advocate for a proactive, inclusive, and ongoing approach to social risk screening and resource connection. Future efforts should prioritize embedding social care into routine clinical workflows to ensure timely and equitable support for all AYA patients.

## Notes

Supplement 1. **eMethods.** Social Determinants of Health Screening Items and Data Analysis **eFigure 1. **Kaiser Permanente Participant Selection **eFigure 2. **Prevalence of Social Risks Among the Total Sample and by AYA Patients With and Without a History of Cancer **eFigure 3. **Forest Plot of Social Risk Associations **eTable 1. **Model Improvements and Odds Ratios for the Association of Any Social Risk and AYA Cancer History **eTable 2. **Association of Any Social Risk and AYA Patients With and Without a History of Cancer Among Those Aged 18 Years or Older **eTable 3. **Cancer Characteristics Risk Factors of Any Social Risk Among AYA Patients With Cancer Aged 18 Years or Older **eTable 4. **Risk Ratios of Any Social Risk and AYA Patients With and Without a History of Cancer **eTable 5. **Comparison of Unweighted and Overlap-Weighted Logistic Regression Results

Supplement 2.
Data Sharing Statement
