# Supplementary material for: Social Risk Prevalence in Adolescent and Young Adult Patients With and Without a History of Cancer
Source: JAMA Netw Open. 2026 Mar 2;9(3):e260244. doi: 10.1001/jamanetworkopen.2026.0244 (PMC12954543; doi:10.1001/jamanetworkopen.2026.0244)
Supplement: Supplement 1. — eMethods. Social Determinants of Health Screening Items and Data Analysis eFigure 1. Kaiser Permanente Participant Selection eFigure 2. Prevalence of Social Risks Among the Total Sample and by AYA Patients With and Without a History of Cancer eFigure 3. Forest Plot of Social Risk Associations eTable 1. Model Improvements and Odds Ratios for the Association of Any Social Risk and AYA Cancer History eTable 2. Association of Any Social Risk and AYA Patients With and Without a History of Cancer Among Those Aged 18 Years or Older eTable 3. Cancer Characteristics Risk Factors of Any Social Risk Among AYA Patients With Cancer Aged 18 Years or Older eTable 4. Risk Ratios of Any Social Risk and AYA Patients With and Without a History of Cancer eTable 5. Comparison of Unweighted and Overlap-Weighted Logistic Regression Results [file jamanetwopen-e260244-s001.pdf]

Supplemental Online Content

Ochoa-Dominguez CY, Mosen DM, Miller KA, et al. Social risk prevalence in adolescent and young adult patients with and without a history of cancer. *JAMA Netw Open*. 2026;9(3):e260244. doi:10.1001/jamanetworkopen.2026.0244

eMethods. Social Determinants of Health Screening Items and Data Analysis

eFigure 1. Kaiser Permanente Participant Selection

eFigure 2. Prevalence of Social Risks Among the Total Sample and by AYA Patients With and Without a History of Cancer

eFigure 3. Forest Plot of Social Risk Associations

eTable 1. Model Improvements and Odds Ratios for the Association of Any Social Risk and AYA Cancer History

eTable 2. Association of Any Social Risk and AYA Patients With and Without a History of Cancer Among Those Aged 18 Years or Older

eTable 3. Cancer Characteristics Risk Factors of Any Social Risk Among AYA Patients With Cancer Aged 18 Years or Older

eTable 4. Risk Ratios of Any Social Risk and AYA Patients With and Without a History of Cancer

eTable 5. Comparison of Unweighted and Overlap-Weighted Logistic Regression Results

This supplemental material has been provided by the authors to give readers additional information about their work.

© 2026 Ochoa-Dominguez CY et al. *JAMA Network Open*.

eMethods. Social Determinants of Health Screening Items and Data Analysis

Social Determinants of Health Screening Items

1. Financial Strain (1 item)
How hard is for you to pay for the very basics like food, housing, medical care and heating? _ Very Hard _Hard _ Somewhat hard _Not very hard _Not hard at all

2. Food Insecurity (2 items)
Within the past 12 months, you worried that your food would run out before you got money to buy more.
_ Never true _Sometimes true _ Often true

Within the past 12 months, the food you bought didn't last and you didn't have money to get more.
_ Never true _Sometimes true _ Often true

3. Housing Instability (3 items)
In the past 12 months, was there a time when you were not able to pay the mortgage or rent on time?
_ Yes _ No

In the last 12 months, how many places have you lived?
_ 1 _2 _ 3 or more

In the past 12 months, was there a time when you did not have a steady place to sleep or slept in a shelter (including now) ?
_ Yes _ No

4. Transportation Needs (2 items)
In the last 12 months, has lack of transportation kept you from medical appointments or from getting medications?
_ Yes _ No

In the last 12 months, has lack of transportation kept you from meetings, work or from getting things needed for daily living?
_ Yes _ No

Data Analysis

We examined several sensitivity analyses. First, we examined an alternative specification of the social risk measure to assess the robustness of our findings. We estimated a modified Poisson regression model treating social risk as a count variable (range: 0–4), representing the total count of social risk domains endorsed by each patient. This specification tested whether modeling social risk as a linear count measure produced results consistent with our primary analysis. This

© 2026 Ochoa-Dominguez CY et al. *JAMA Network Open*.

alternative model demonstrated the findings were directionally consistent with our main results, providing support for the stability of our conclusions. However, because the Poisson regression model treating social risk as a count variable (range: 0–4), showed evidence of overdispersion (Pearson χ^2/df = 1.62), suggesting that the variance exceeded the mean. Given this violation of the Poisson assumption, we estimated a Negative Binomial regression model that includes a dispersion parameter to account for overdispersion. The Negative Binomial model demonstrated good fit, with Pearson χ^2/df = 0.94 and Deviance/df = 0.82, indicating that overdispersion present in the Poisson model was adequately addressed. The estimated dispersion parameter (α = 1.31; 95% CI: 1.27–1.35) was significantly greater than zero, confirming that the Negative Binomial distribution provided a more appropriate representation of the variance structure. Second, we conducted a sensitivity analysis using the modified Poisson regression, which included cancer history, response year, and age. The results were similar to our logistic regression model results. Third, we conducted a sensitivity analysis using overlap propensity score weighting with robust error variance. Specifically, we trimmed the analytic sample to the range of raw propensity scores observed among AYA cancer patients to ensure appropriate covariate overlap between groups and to reduce potential bias from non-comparable observations. This approach increases the stability of the estimates by down-weighting individuals with extreme propensity scores who contribute disproportionately to variance. The results of this sensitivity analysis were consistent with our primary findings, supporting the validity of our conclusions.

© 2026 Ochoa-Dominguez CY et al. *JAMA Network Open*.

eFigure 1. Kaiser Permanente Participant Selection

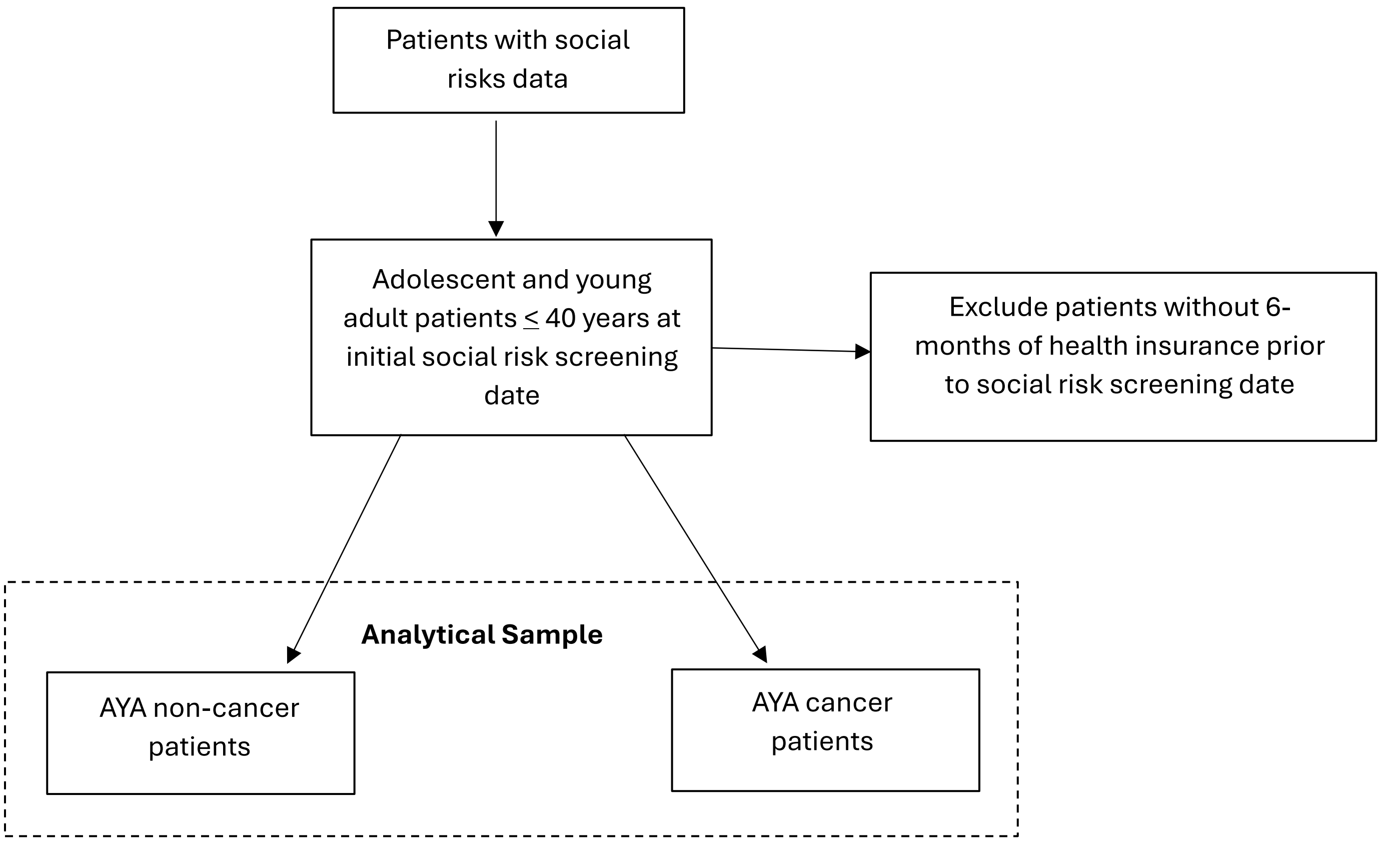

© 2026 Ochoa-Dominguez CY et al. *JAMA Network Open*.

eFigure 2. Prevalence of Social Risks Among the Total Sample and by AYA Patients With and Without a History of Cancer

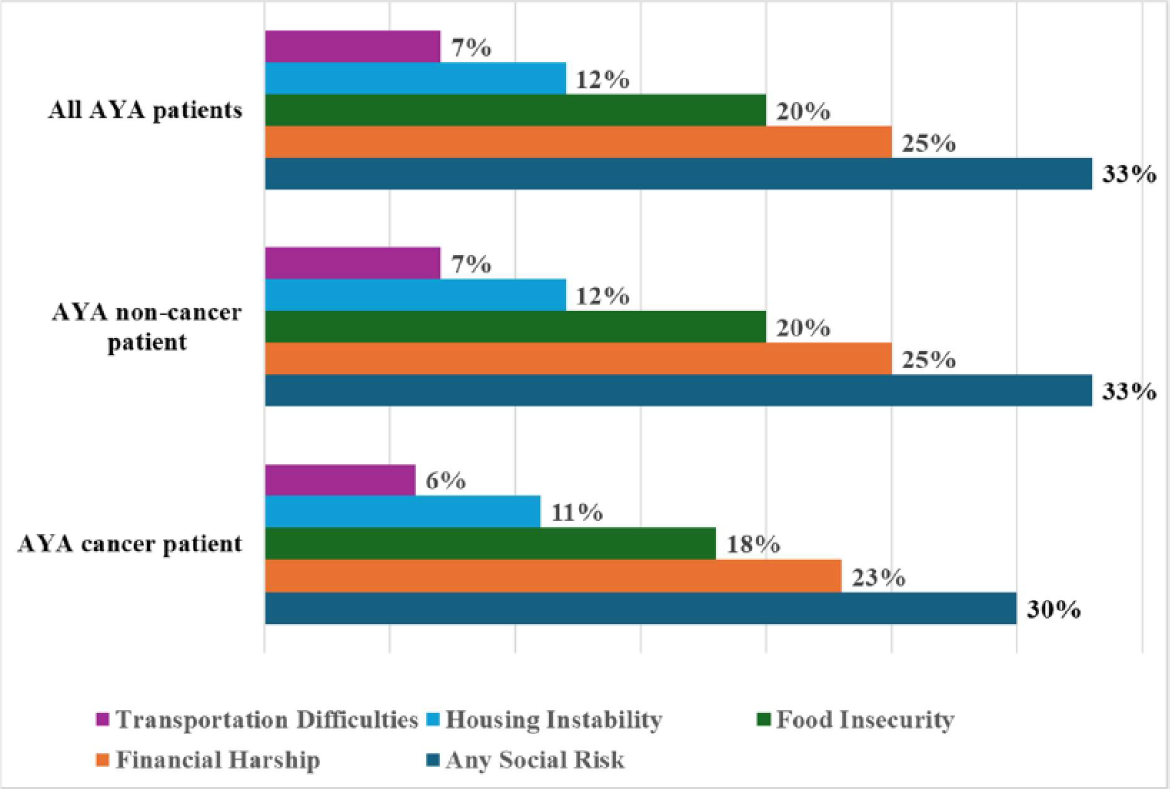

Variables	Total Sample (n=96,127)	Non-Cancer Patients (n=94,888)	Cancer Patients (n=1,239)
**Social risk category**			
Financial hardship			
Yes	23,858 (25%)	23, 570 (25%)	288 (23%)
Food insecurity			
Yes	18,937 (20%)	18,719 (20%)	218 (18%)
Housing instability			
Yes	11,628 (12%)	11,488 (12%)	140 (11%)
Transportation difficulties			
Yes	6,908 (7%)	6,825 (7%)	77 (6%)
**Any Social Risk**			
≥ 1 Social risk	31,659 (33%)	31,287 (33%)	372 (30%)

© 2026 Ochoa-Dominguez CY et al. *JAMA Network Open*.

eFigure 3. Forest Plot of Social Risk Associations

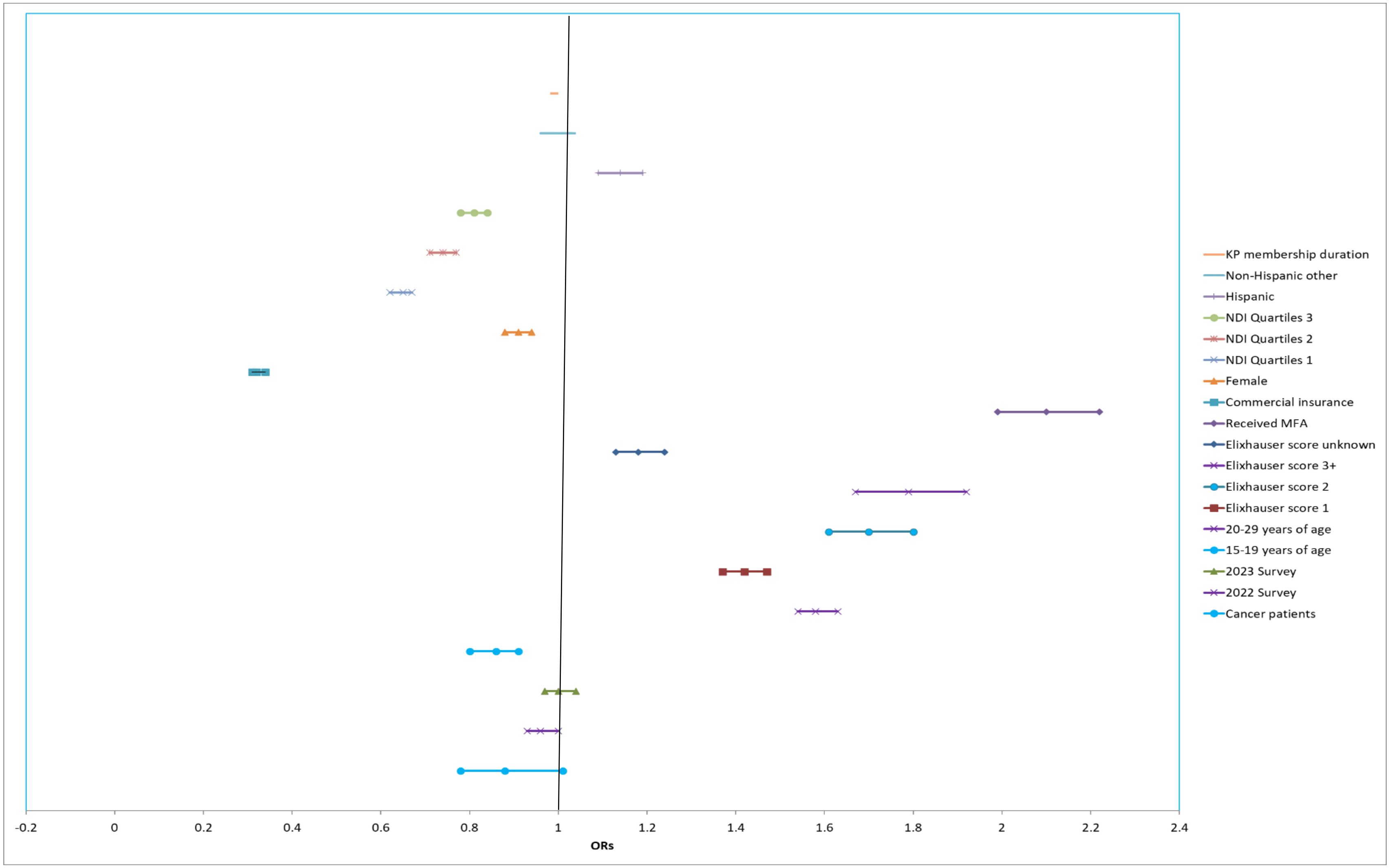

© 2026 Ochoa-Dominguez CY et al. *JAMA Network Open*.

eTable 1. Model Improvements and Odds Ratios for the Association of Any Social Risk and AYA Cancer History

**Models**	**AIC**	**-2 Log L**	**C-Statistic**	**AYA Cancer Patient OR (95% CI)**
Model 1 (Baseline)	121833.20	121829.20	0.501	0.872 (0.772 – 0.986)
Model 2 (+ Survey Year)	121827.87	121819.87	0.506	0.876 (0.775 – 0.990)
Model 3 (+ Age Category)	120743.31	120731.31	0.561	0.960 (0.849 – 1.085)
Model 4 (+ Comorbidities)	119359.10	119339.10	0.596	0.904 (0.799 – 1.024)
Model 5 (+ MFA)	118580.63	118558.63	0.610	0.848 (0.748 – 0.961)
Model 6 (+ Health Insurance)	114508.65	114484.65	0.663	0.820 (0.721 – 0.932)
Model 7 (+ Sex)	114268.73	114242.73	0.663	0.832 (0.732 – 0.946)
Model 8 (+ NDI 2020)	113721.22	113689.22	0.672	0.833 (0.732 – 0.947)
Model 9 (+ Race/Ethnicity)	113672.85	113636.85	0.674	0.835 (0.734 – 0.951)
Model 10 (+ KP Membership duration)	113088.96	113050.96	0.679	0.882 (0.775 – 1.005)

Abbreviations. MFA: Medical Financial Assistance; NDI: Neighborhood Deprivation Index.
Notes: *Model 1* = Cohort and Outcome, *Model 2* = Cohort + Survey Year and Outcome, *Model 3* = Cohort + Survey Year + Age and Outcome, *Model 4* = Cohort + Survey Year + Age + Elixhauser score and Outcome, *Model 5* = Cohort + Survey Year + Age + Elixhauser score + Medical financial assistance and Outcome, *Model 6* = Cohort + Survey Year + Age + Elixhauser score + Medical financial assistance + Health insurance and Outcome, *Model 7* = Cohort + Survey Year + Age + Elixhauser score + Medical financial assistance + Health insurance + Sex and Outcome, *Model 8* = Cohort + Survey Year + Age + Elixhauser score + Medical financial assistance + Health insurance + Sex + NDI 2020 and Outcome, *Model 9* = Cohort + Survey Year + Age + Elixhauser score + Medical financial assistance + Health insurance + Sex + NDI 2020 + Race/ethnicity and Outcome, *Model 10* = Cohort + Survey Year + Age + Elixhauser score + Medical financial assistance + Health insurance + Sex + NDI 2020 + Race/ethnicity + KP membership duration and Outcome.

© 2026 Ochoa-Dominguez CY et al. *JAMA Network Open*.

eTable 2. Association of Any Social Risk and AYA Patients With and Without a History of Cancer Among Those Aged 18 Years or Older

	Any Social Risk			
	Unadjusted OR [95% CI]	P[1]	Adjusted OR [95% CI]	P[1]
Cancer patients (ref=non-cancer patients)	0.875 [0.775, 0.989]	**0.0330**	0.887 [0.779, 1.011]	0.0719
2022 Survey (ref=2024)	0.948 [0.916, 0.982]	**0.0032**	0.965 [0.930, 1.002]	0.0627
2023 Survey (ref=2024)	0.960 [0.929, 0.992]	**0.0159**	1.004 [0.969, 1.040]	0.8265
15-19 years of age (ref=30-40)	0.788 [0.740, 0.839]	**<0.0001**	0.866 [0.810, 0.926]	**<0.0001**
20-29 years of age (ref=30-40)	1.543 [1.500, 1.587]	**<0.0001**	1.582 [1.535, 1.630]	**<0.0001**
Elixhauser score 1 (ref=0)	1.505 [1.454, 1.559]	**<0.0001**	1.418 [1.367, 1.471]	**<0.0001**
Elixhauser score 2 (ref=0)	1.928 [1.830, 2.031]	**<0.0001**	1.699 [1.608, 1.795]	**<0.0001**
Elixhauser score 3+ (ref=0)	2.240 [2.097, 2.394]	**<0.0001**	1.791 [1.669, 1.922]	**<0.0001**
Elixhauser score Unknown (ref=0)	1.263 [1.211, 1.317]	**<0.0001**	1.183 [1.132, 1.235]	**<0.0001**
Received MFA (ref=No MFA)	2.258 [2.143, 2.380]	**<0.0001**	2.105 [1.991, 2.224]	**<0.0001**
Commercial (ref=Other)	0.286 [0.276, 0.297]	**<0.0001**	0.323 [0.311, 0.335]	**<0.0001**
Female (ref=Male)	1.020 [0.992, 1.049]	0.1729	0.906 [0.879, 0.933]	**<0.0001**
NDI Quartiles 1 (ref=4)	0.558 [0.537, 0.580]	**<0.0001**	0.650 [0.624, 0.677]	**<0.0001**
NDI Quartiles 2 (ref=4)	0.660 [0.636, 0.686]	**<0.0001**	0.736 [0.708, 0.766]	**<0.0001**
NDI Quartiles 3 (ref=4)	0.747 [0.719, 0.776]	**<0.0001**	0.817 [0.785, 0.849]	**<0.0001**
Hispanic (ref=Non-Hispanic White)	1.383 [1.329, 1.439]	**<0.0001**	1.143 [1.095, 1.192]	**<0.0001**
Non-Hispanic Other (ref=Non-Hispanic White)	1.052 [1.018, 1.086]	**0.0025**	0.995 [0.961, 1.030]	0.7751
KP membership duration	0.997 [0.997, 0.997]	**<0.0001**	0.997 [0.997, 0.998]	**<0.0001**

Abbreviations. OR: Odds Ratio; CI: Confidence Interval; MFA: Medical Financial Assistance; NDI: Neighborhood Deprivation Index
Notes: [1] P values are two-sided.

© 2026 Ochoa-Dominguez CY et al. *JAMA Network Open*.

eTable 3. Cancer Characteristics Risk Factors of Any Social Risk Among AYA Patients With Cancer Aged 18 Years or Older

	**Any Social Risk**			
	Unadjusted OR [95% CI]	P[1]	Adjusted OR [95% CI]	P[1]
**Age at diagnosis**				
15-19 (ref=30-39)	0.954 [0.536, 1.696]	0.8718	1.270 [0.688, 2.344]	0.4449
20-29 (ref=30-39)	0.920 [0.705, 1.200]	0.5384	1.109 [0.817, 1.505]	0.5059
**Time since diagnosis**				
<1 year (ref= 5+ years)	1.138 [0.813, 1.594]	0.4513	1.447 [0.943, 2.219]	0.0906
1-5 years (ref= 5+ years)	1.384 [1.044, 1.834]	**0.0239**	1.450 [1.002, 2.098]	**0.0486**
**Cancer site[2]**				
Reproductive (ref=other sites)	1.006 [0.625, 1.619]	0.9794	1.185 [0.676, 2.075]	0.5535
Solid (ref=other sites)	0.699 [0.420, 1.162]	0.1668	0.704 [0.392, 1.264]	0.2398
Hematologic (ref=other sites)	1.230 [0.656, 2.309]	0.5189	1.607 [0.768, 3.361]	0.2078
**Tumor stage[3]**				
0 (ref=Unknown/Unstaged)	0.682 [0.487, 0.956]	**0.0263**	0.824 [0.538, 1.263]	0.3745
1 (ref=Unknown/Unstaged)	0.800 [0.583, 1.096]	0.1642	1.064 [0.722, 1.568]	0.7542
2 (ref=Unknown/Unstaged)	0.860 [0.505, 1.466]	0.5799	0.819 [0.455, 1.475]	0.5059
3 (ref=Unknown/Unstaged)	0.906 [0.545, 1.506]	0.7044	1.065 [0.607, 1.869]	0.8255
4 (ref=Unknown/Unstaged)	0.712 [0.338, 1.501]	0.3722	0.600 [0.241 1.496]	0.2730

Abbreviations. OR: Odds Ratio;CI: Confidence Interval.

Notes: [1] P values are two-sided.

[2] Cancer site: Reproductive system (breast, uterine cervix, corpus and uterus, male genital system), Solid tumors (colorectal & rectum, skin, endocrine system, brain and other nervous system), Hematologic (Leukemia and Lymphoma), Other (digestive system, eye and orbit, bones and soft tissues, oral cavity and pharynx, respiratory system, urinary system, digestive system, and unknown).

[3] Tumor stage: Unknown/Unstaged - Includes patients not assigned a tumor stage at diagnosis or for whom stage information at time of diagnosis was unavailable.

© 2026 Ochoa-Dominguez CY et al. *JAMA Network Open*.

eTable 4. Risk Ratios of Any Social Risk and AYA Patients With and Without a History of Cancer

	RR [95% CI]	P[1]
Cancer patients (ref=non-cancer patients)	0.91 [0.82, 1.00]	0.0575
2022 Survey (ref=2024)	1.01 [0.98, 1.04]	0.4788
2023 Survey (ref=2024)	1.03 [1.00, 1.06]	0.0523
15-19 years of age (ref=30-40)	0.88 [0.84, 0.93]	<0.0001
20-29 years of age (ref=30-40)	1.42 [1.38, 1.45]	<0.0001
Elixhauser score 1 (ref=0)	1.33 [1.29, 1.37]	<0.0001
Elixhauser score 2 (ref=0)	1.53 [1.47, 1.60]	<0.0001
Elixhauser score 3+ (ref=0)	1.61 [1.53, 1.69]	<0.0001
Elixhauser score Unknown (ref=0)	1.15 [1.11, 1.19]	<0.0001
Received MFA (ref=No MFA)	1.68 [1.61, 1.75]	<0.0001
Commercial (ref=Other)	0.43 [0.41, 0.44]	<0.0001
Female (ref=Male)	0.95 [0.93, 0.98	<0.0001
NDI Quartiles 1 (ref=4)	0.72 [0.70, 0.74]	<0.0001
NDI Quartiles 2 (ref=4)	0.79 [0.77, 0.82]	<0.0001
NDI Quartiles 3 (ref=4)	0.87 [0.84, 0.89]	<0.0001
Hispanic (ref=Non-Hispanic White)	1.13 [1.09, 1.17]	<0.0001
Non-Hispanic Other (ref=Non-Hispanic White)	1.02 [1.00, 1.05]	0.0933
KP membership duration	0.99 [0.99, 0.99]	<0.0001

Abbreviations. RR: Risk Ratio; CI: Confidence Interval; MFA: Medical Financial Assistance; NDI: Neighborhood Deprivation Index

Notes: [1] P values are two-sided

© 2026 Ochoa-Dominguez CY et al. *JAMA Network Open*.

eTable 5. Comparison of Unweighted and Overlap-Weighted Logistic Regression Results

**Model**	**Odds Ratio**	**95% CI**	**P Value**
**Unweighted Model**	0.88	0.78–1.01	0.059
**Overlap-Weighted Model**	0.90	0.76–1.07	0.251

© 2026 Ochoa-Dominguez CY et al. *JAMA Network Open*.
