# Supplementary material for: Social Risk Prevalence in Adolescent and Young Adult Patients With and Without a History of Cancer
Source: JAMA Netw Open. 2026 Mar 2;9(3):e260244. doi: 10.1001/jamanetworkopen.2026.0244 (PMC12954543; doi:10.1001/jamanetworkopen.2026.0244)
Supplement: Supplement 2. — Data Sharing Statement [file jamanetwopen-e260244-s002.pdf]

Data Sharing Statement

Data
Data available: No

Additional Information
Explanation for why data not available: Data from Kaiser Permanente are protected and only accessible via an approved data access request. Thus, we will not be making these data available outside of this structure.
